# Just and inclusive end-of-life decision-making for long-term care home residents with dementia: a qualitative study protocol

**DOI:** 10.1186/s12904-022-01097-x

**Published:** 2022-11-22

**Authors:** N. Sutherland, O. St. Amant, S. Dupuis, P. Kontos, E. Wiersma, M. Brennan

**Affiliations:** 1 School of Nursing, Lakehead University, Centre for Education and Research on Aging and Health, Lakehead University, 955 Oliver Road, Thunder Bay, Ontario, P7B 5E1 Canada; 2 Daphne Cockwell School of Nursing, Toronto Metropolitan University, 350 Victoria Street, Toronto, Ontario M5B 2K3 Canada; 3 Department of Recreation and Leisure Studies, University of Waterloo, 200 University Avenue, Waterloo, Ontario, N2L 3G1 Canada; 4 KITE Research Institute, Toronto Rehabilitation Institute - University Health Network, Dalla Lana School of Public Health, University of Toronto, 550 University Avenue, Toronto, Ontario M5G 2A2 Canada; 5 Department of Health Sciences, Lakehead University, Centre for Education and Research on Aging and Health, Lakehead University, 955 Oliver Road, Thunder Bay, Ontario, P7B 5E1 Canada; 6 Bora Laskin Faculty of Law, Lakehead University, 955 Oliver Road, Thunder Bay, Ontario, P7B 5E1 Canada

**Keywords:** People living with dementia, Long-term care homes, Palliative care, End-of-life decision-making

## Abstract

**Background:**

Many people living with dementia eventually require care services and spend the remainder of their lives in long-term care (LTC) homes. Yet, many residents with dementia do not receive coordinated, quality palliative care. The stigma associated with dementia leads to an assumption that people living in the advanced stages of dementia are unable to express their end-of-life needs. As a result, people with dementia have fewer choices and limited access to palliative care. The purpose of this paper is to describe the protocol for a qualitative study that explores end-of-life decision-making processes for LTC home residents with dementia.

**Methods/design:**

This study is informed by two theoretical concepts. First, it draws on a relational model of citizenship. The model recognizes the pre-reflective dimensions of agency as fundamental to being human (irrespective of cognitive impairment) and thereby necessitates that we cultivate an environment that supports these dimensions. This study also draws from Smith’s critical feminist lens to foreground the influence of gender relations in decision-making processes towards palliative care goals for people with dementia and reveal the discursive mediums of power that legitimize and sanction social relations.

This study employs a critical ethnographic methodology. Through data collection strategies of interview, observation, and document review, this study examines decision-making for LTC home residents with dementia and their paid (LTC home workers) and unpaid (family members) care partners.

**Discussion:**

This research will expose the embedded structures and organizational factors that shape relationships and interactions in decision-making. This study may reveal new ways to promote equitable decision-making towards palliative care goals for LTC home residents with dementia and their care partners and help to improve their access to palliative care.

## Introduction

There is an urgent need for contextual understandings and theoretical advancements regarding end-of-life for people living with dementia [1, 2]. Although most die in long-term care (LTC) homes [3, 4], residents’ end-of-life experiences have been reported to be suboptimal, with aggressive over or undertreatment often leading to physical and emotional distress, or transfers to acute care centres with residents dying outside of familiar spaces [5–9]. In such cases, LTC home residents with dementia may be denied palliative care, an approach focused on alleviating suffering and promoting quality of life [10].

The quality of life and death for people living with dementia is significantly shaped by decision-making about nutrition, activity, and recurrent infections in the last 6 to 18 months of life [5, 6, 11]. The stigma associated with taken-for-granted assumptions that people with dementia lose their selfhood, and their ability to know and express their needs [2, 12], has frequently led health care providers to negate the preferences of people with dementia [13–18]. The medical emphasis on physical and mental deficits [13–18], in addition to paternal beneficence and a narrow focus on reducing risks [19], may result in imposing decisions that threaten residents’ citizenry rights and sacrifice their quality of life (e.g., sitting outdoors).

Current palliative care policies focus on interventions early in the diagnosis. However, these advance care plans (ACPs) —structured discussions to make known future goals of care in case of mental incapacity [20]—are problematic for people with dementia. Notable issues include the timing of professionals to introduce ACPs and a reluctance of people with dementia and family members to discuss the future [20–23]. While helpful to some [20, 24], ACPs can at times be irrelevant where they lack context of the immediate situation [20, 25] and create ethical dilemmas about plans not being implemented [26–28]. More poignantly, ACPs fail to reflect the contemporaneous preferences of people with dementia [29]. As such, people living with dementia are severely disadvantaged as they are denied chances to change their minds as they experience decline [29–31]. To address these potential inequities, this study critically explores end-of-life decision-making for LTC home residents with dementia. This paper outlines the protocol for this qualitative study.

## Background

Most of the palliative care research related to people living with dementia has been centred on their lack of decision-making capacity [29] and substitute family decision-making. Yet, little attention has been paid to the relational processes that shape decision-making for residents living with dementia in LTC homes [8, 13, 29]. Few investigators have attempted to facilitate the perspectives of residents with dementia and rarely has research included the perspectives of LTC home residents with advanced dementia [32, 33]. Research has indicted that, given the opportunity, people with dementia in early *and* late stages can express their preferences through repeated narratives and embodied expressions (e.g., crying) and discuss dying without undue distress [32, 33]. Yet, end-of-life experiences of people living with dementia in LTC homes are, for the most part, obtained from family carers and not by people with dementia [29]. Without the inclusion of people living with dementia themselves, there is an inability to fully address their palliative care needs and preferences.

Although the majority of the literature has focused on family decision-makers, there is little understanding of the complexities associated with substitute decision-making as it relates to people living with dementia [29]. There is a gap in knowledge of how power relations of status, ethnicity, religion, or gender shape decision-making experiences of people living with dementia in LTC homes. Although gender equity for family carers has been widely explored, there has been little recognition of the gender of the person with dementia whose diagnosis often supersedes other personal identities such as being a wife, brother, mother, or son [34, 35]. Research has indicated that healthcare workers initiate more interactions with male rather than female residents and often inscribe residents’ gender through superficial acts such as hair styling [36, 37]. Another study found that, compared to their male counterparts, the autonomy of women living with dementia is less likely to be encouraged by their spouses [37]. As decision-making and power relations of gender are strongly linked [38], a gender analysis is essential to understanding end-of-life decision-making men and women with dementia living in LTC homes.

Additionally, LTC home organizational structures have been focused on biomedical aspects of care [13, 30–44] with emphasis placed on measurable assessments and objective knowledge of physicians, and to a lesser extent nurses. The personal knowledge of family and personal support workers (aids), often intimately involved in the care of people with dementia, is most often undervalued and excluded [13, 40–44]. Long-term care home cultures that focus on medical needs and tasks overlook the importance of relational caring [36, 45–47] that recognises the relational embeddedness of all care contexts and privileges the relationship-building fundamental to supporting humane decision-making processes. Relationship-building among residents, family, and staff has been found to be essential to understanding and caring for residents, enhancing quality of life [43] and end-of-life decision-making processes [1, 48, 49]. Organizational barriers to relational models of care and quality palliative care in LTC homes (e.g., heavy workloads, high staff turnover, lack of private spaces, insufficient resources, and lack of physician availability and palliative care policies) have been identified [43, 46, 50–53]. A relational model recognizes the interdependence of care relationships as well as the structural and organizational power relations that shape care [54, 55].

To address structural power relations in end-of-life decision-making, this critical study draws upon the theories of relational citizenship [54, 56] and critical feminism [57, 58]. We first provide a brief description of the underlying concepts of personhood, relational caring, and citizenship, all of which significantly shape the experiences of people living with dementia.

### Personhood to relational care

The predominate biomedical view of dementia as a neurological disorder characterized by a progressive loss of selfhood has tended to focus on the disease and overlook people with dementia and the sociocultural environment that shape their experiences. Kitwood’s [55] definition of personhood, a status bestowed upon people through relationships with others, has been revolutionary to understanding the lived experiences of people with dementia. This perspective suggests that the way family and staff interact with people living with dementia influences the attitudes and actions of people living with dementia. When guided by this perspective, LTC home staff centre their focus of care on the person rather than the disease.

Despite advancements, person-centre care has been narrowly focused on individual needs rather than on the potential of a person with dementia to influence circumstances [29]. This approach overlooks the pre-reflexive capacity of persons with dementia to express their preferences. In an ethnographic study examining people with dementia living in LTC homes [34], Kontos et al. found that residents interacted with intentional purpose and meaning through bodily expressions. For example, often with only bodily movements and gestures, residents with dementia expressed their dislike for food, the importance of jewelry for their self-presentation, or their respect for the etiquette of removing a hat when dining [34]. The inextricable link of one’s bodily dispositions to cultural attitudes and beliefs highlights the pre-reflective capacity of humans to express themselves [34]. Thus, bodily movements and gestures are important sources of selfhood and self-expression and are present irrespective of mental capacity. Accordingly, the biographical history, preferences, values, or cultural dispositions and bodily expressions of the person with dementia must be considered when interpreting interactions and supporting care decisions [34].

Additionally, everyday decision-making for people living with dementia has been found to be an interdependent activity rather than an individual choice [59]. Family and staff members’ personal knowledge of the person with dementia has been found to be important to identify residents’ discomfort or desires and avoid unnecessary transfers to hospital [9, 53]. In a study examining family decision-making for people with advanced dementia, Elliot [48] found that decision-making was often based on storytelling of the person living with dementia. Thus, although people with dementia in LTC homes are often unable to voice complaints [2], LTC home staff and family can support decision-making that includes the preferences and needs of people living with dementia.

This relational approach that accounts for interdependency in relationships also emphasizes the structures that shape LTC home interactions. Structures are the dominant cultural and political discourses that construct, prescribe, and regulate care relations and experiences [47, 57]. Thus, in using a relational lens, decision-making related to care is focused beyond the dyad of care-recipient and care-giver, to all the people (e.g., housekeeping staff, personal support workers [PSWs]) and the structures (e.g., staff workload) involved in shaping a supportive and inclusive environment in the LTC home.

### Relational caring to relational citizenship

To bring in an inclusive approach, we employ the lens of citizenship. Traditionally, citizenship has been conceptualized as a status proffered for people to be treated the same as their fellow citizens. This conceptualization assumes that people have the physical and mental capacity to exert their rights and leaves out those who are unable to advocate for themselves [60]. Such an understanding discriminates against people with cognitive impairment who are unable to claim rights [60, 61]. Thus, citizenship status overlooks differences in power that shape how people are perceived, treated, and presented with opportunities to make choices.

More recently, the status of citizenship has been viewed as a practice in which an optimal community and environment are provided in which people can exercise their rights [61]. Thus, a citizenship lens would be focused on the macro level of how the LTC home is structured institutionally and culturally and how these structures shape relationships and roles between and among people and their sociopolitical environment. At the meso and micro levels, the application of this social justice lens focuses, for example, on how LTC home staff and residents’ family members foster an environment in which the person with dementia can be included in decisions about their lives. This approach allows for an analysis of power and a path to challenge discriminatory practices [60, 61]. As decision-making is an important enactment of citizenship, using a citizenship lens is highly suitable to examine decision-making for LTC home residents with dementia [60].

With an interest in utilizing both concepts of relationality and citizenship, this study employs Kontos’s theory of relational citizenship [54, 55] and a critical feminist lens [57, 58] to challenge how broader institutional, governmental, or sociocultural factors shape experiences of decision-making (citizenship) and potentially discriminate against LTC home residents with dementia and their care partners.

### Relational citizenship

The core theoretical tenet of the relational model of citizenship [54] is embodied selfhood [34, 36, 54, 62–64] which considers both the pre-reflective intentionality of the body and its natural (pre-social) engagement with the world (the body’s power of natural expression), as well as the ongoing socio-cultural relationship between the pre-reflective body and the world (history, culture, power, and discourse) [34, 36, 54, 62–64]. A core assumption with this model is that embodied selfhood is fundamental to the human condition, and thus it is essential that it is supported through socio-political institutions and organizational practices at the local level of citizenship. The model is thus furnished with a human rights ontology that recognizes these pre-reflective dimensions of agency as fundamental to being human (irrespective of cognitive impairment) and thereby necessitates that we cultivate a relational environment that supports these dimensions to the fullest extent possible [54, 56, 65].

### Critical feminist lens

We also employ Smith’s critical feminist lens [57, 58] to foreground how gender shapes end-of-life decision-making processes for people living with dementia. According to Smith, discourses, speech, or written texts, dictate how we present ourselves or perceive others, the actions we take and the events of which we partake. Emanating from dominant groups - diffuse networks of institutions (e.g., familial or governmental) - these discourses are abstract ideas and mediums of power that legitimize relationships and roles [57, 58]. Smith’s gender lens starts with women’s everyday embodied lives to explicate how these complex social relations that constrain or exclude are (re)produced. This critical feminist lens is consistent with Kontos et al.’s relational model of citizenship [54, 63] and Connell’s [66] concept of gender as relational, interacting at multiple levels that involve the personal, interpersonal, organizational and socio-political dimensions. As gender does not operate in isolation, a gender analysis must also consider how other social relations (e.g., race and class) may interact with gender [57, 58]. Moreover, given that care in LTC homes is predominately given and received by women, entering with a gender lens is appropriate [67] to an analysis that explores just practices in LTC homes.

By employing the theoretical perspectives of relational citizenship and critical feminism, this study will provide an in-depth, comprehensive understanding of the embedded social structures and organizational factors shaping end-of-life decision-making for LTC home residents with dementia and their care partners. This study is guided by the following research questions: 1) How are end-of-life decisions made for residents with dementia in LTC homes? 2) How do gender and other broader social structures (e.g., race and class) shape decision-making? 3) How do organizational factors (e.g., material and economic resources) affect decision-making? 4) What strategies can be used to promote just and inclusive end-of-life decision-making for residents with dementia and their care partners (unpaid family members and paid LTC home workers)?

## Methods

### Study design

This study employs a critical ethnographic design [68]. Aligned with a critical feminist lens, critical ethnography aims to connect everyday meanings and experiences to broader structures of power [68]. Given the presumed loss of selfhood associated with people living with dementia, it is important to illuminate and challenge the structural factors that shape power relations and potentially constrain or exclude their participation related to end-of-life decision-making processes. This critical study aims to shed light on policies, everyday attitudes, practices, and ways of relating that shape end-of-life decision-making processes for LTC home residents with dementia and their care partners.

### Recruitment and sample

We are partnering with three diverse LTC homes in Ontario, Canada for which the researchers have established relationships. Located in urban centres, one is a public not-for-profit (150 beds) and another is a larger private not-for-profit home (543 beds). The third LTC home is a small, private for-profit home (25 beds). Recruiting from three diverse LTC homes provides an understanding of end-of-life decision-making in different care contexts and facilitates analysis of differences across the sites. As an example, private for-profit LTC homes have reported fewer hours of care and lower nursing staff levels, factors associated with poorer health outcomes for LTC home residents [69]. Given the complexity of the diverse sites, we will recruit 9 to 12 residents (3 to 4 at each site). For each resident, we will recruit 3 to 4 of their care partners (family members or LTC home staff and other healthcare workers), potentially involving a total of 60 participants, which is consistent with the recommended 30 to 50 participants in ethnographic studies [70, 71].

This study is engaging three key stakeholder groups: 1) residents with dementia; 2) unpaid care partners (family members); and 3) paid LTC home care partners such as PSWs, nurses, unit managers, leisure or dietary staff, for example. As this study is focused on how gender and other social relations affect decision-making, we will include, as much as possible, an equal number of male and female residents with dementia, and paid and unpaid care partners, and representation by status (e.g., nurses, PSWs) or ethnicity [70].

### Data collection

Upon gaining entry, we will familiarize ourselves within the LTC homes. To illuminate dynamic end-of-life decision-making processes, ethnographic methods of interview, observation, and document review will be employed.

#### Interviews

We will be interviewing residents with dementia, paid (LTC home workers), and unpaid care partners (family members). By engaging residents with dementia face-to-face, we may be able to identify practices to support people with dementia in decision-making processes and explore how they are treated (or not) as full citizens. As much as possible, we will employ, based on the literature, best practice strategies that include people living with dementia.

#### Engaging the person with dementia and ethical considerations

We will be approaching each person with dementia as a unique person with individual desires and needs [72–75]. When a potential person with dementia is identified, we will first acquire background information by asking family and staff about the biographical background of the person with dementia and the current opinions and cognitive tests related to capacity. If the person with dementia is unable to give consent, we will be asking for consent from the substitute decision maker. With consent by proxy, we will be using the ethical approach recommended when working with people with dementia and engage in ‘process consent’ for each visit [76]. Consistent with the concept of embodied selfhood [34, 36, 54] we will be attentive and responsive to how the person with dementia expresses reluctance, objection, or willingness. We will observe for verbal, non-verbal responses, and implied meanings. We will assess how the person with dementia likes to interact and look for meaningful ways to communicate (e.g., use eye contact, touch, or images) [73, 74]. We will reflect on our own attitudes and body language and how these affect interactions. If the resident expresses discomfort with researchers, we will withdraw and return to the resident at another time. If the resident continues to show discomfort, we will not include the resident in the study.

We will employ strategies drawn from the literature to encourage people living with dementia to talk about their experiences. We will take the time to develop relationships and engage during times most suitable to participants [73–75]. We will use short and frequent interviews and use an interactive interview style [76] in which we will use prompts and cues from the local environment and focus on what matters to participants. For example, in an activity we may ask, “Why do you like this activity?” We will start with a general warm up question that can be repeated [72–74]: “What is it like living here?”. This open-ended question permits participants to draw attention to their own concerns. We will ask thematic questions related to the inquiry [76]; for example, we will ask: “Tell me about the people who care for you”; “Who makes decisions about your care?”; What would it this be like if you were a man/woman?”; “Have you thought about dying?” [32, 33].

Additionally, we will be interviewing care partners of residents with dementia, including family members and LTC home workers. The purpose of these interviews is to explore how care partners perceive end-of-life decision-making processes and their role in those processes; how they view residents, family, and other staff members; how they perceive they are viewed by others; and how gender and other broader structures and organizational factors shape decision-making processes. For example, we will ask: “Can you tell me about a time when a decision was made?; How were you supported?; How does being a man or a woman (wife or daughter) affect your interactions? All individual interviews will be audio-recorded and transcribed.

#### Observations

Being present in the natural environment and attentive to contradictions and non-verbal expressions will afford insight into interactions, processes, and contexts related to decision-making [77, 78]. Observations will also be critical to understanding people with dementia in LTC homes. To explore resident-family-LTC home paid care partner interactions, we will be guided by Kontos’s concept of embodied selfhood [34, 36, 54]. We will be attentive to how the person with dementia may use different modes of expression such as words, postures, gestures, movements, and will interpret this information with reference to the resident’s biography, life history, and family and staff’s knowledge of the resident’s likes or dislikes and personal modes of communicating preferences [34, 36, 54, 79].

Based on the relational model of citizenship [54], we will observe if the person with dementia is treated with dignity and supported in her/his engagement with others and in self-expression [34, 36, 54, 80–82]. Employing a critical feminist lens [50, 58] we will consider whether the person with dementia is accepted and treated with respect, free from stigmatizing and discriminating practices. We will examine if the person with dementia is comfortable, in a tranquil environment in which emotional and spiritual needs are met, and where they are free from pain and suffering [80, 83]. We will also explore the ways in which bodily expressions and the socio-cultural background of the person with dementia is considered and included in end-of-life decision-making [34, 36, 54], and whether decisions are consistent with their personal expressions and background.

In alignment with ethnographic studies [78], we will spend time in the LTC homes observing everyday interactions between and among the person with dementia and their care partners. We will observe places where decisions are made, which will include care conferences (e.g., resident-family-paid care worker meetings to plan care goals) and other staff gatherings (e.g., shift reports, physician or allied health visits). We will also observe times when the resident, family, or LTC home workers interact about care decisions to reveal how people are included or excluded or how people enact, acquiesce, or resist power. These care decisions may occur during meal or leisure times related to making decisions about nutritional intake or activity involvement. We will spend intensive periods of 5-7 hours per week, during different times (day or evening), for 12-14 months (considered prolonged observation) [84] or until there is an in-depth understanding of end-of-life discussions and how decisions are made. We will assume a peripheral role in which we will participate as volunteers in public activities at meal and leisure times, however, we will not fully participate in direct care activities [77, 78]. We will notify LTC home care workers about the study by posting notices and giving short presentations at staff, family, and resident council meetings to describe the purpose of the study, who and what is involved, and how care partners could be involved. Our focus during observations will always be on the individuals who have consented to participate in the study.

### Review of documents

Documents will be analyzed to gain an understanding of the structural and organizational influences on everyday decision-making interactions in LTC homes. We will review documents related to LTC home agency standards and practices (e.g., current governmental and organizational policy and practice documents, educational material used for staff training, information pamphlets for residents/family members), professional documents that guide practices (e.g., College of Nurses of Ontario, Ontario Medical Association) and regulations pertaining to advance directives. In addition, we will ask study participants to identify documents they feel are relevant. We will review only the resident’s documented advanced directives or future wishes, provided there is consent.

### Data analysis

For a critical analysis of interview and observational data, and with reference to the relational model of citizenship informing our study, we will use a voice-centred relational approach [85] guided by four analytical reviews: 1) We will acquire an overall picture of decision-making processes while reflecting on our biases and assumptions; 2) We will focus on how participants use the pronoun “I” to identify how they view themselves 3) We will focus on the pronouns “they” to view how participants relate to others in organizational and structural power relations; and, 4) we will link micro-level individual attitudes and behaviours to broader institutional, cultural, and societal discourses. Using a critical feminist lens [57, 58] we will explore how gender is expressed or depicted and how gender intersects with other social relations to shape decision-making. We will examine how power relations are exerted and effected, asking: Who benefits? Who is disadvantaged? Whose interests are served? Who is controlling? Who is resisting? [57, 58, 86]. Aligned with our theoretical frameworks, we will analyze how participants’ attitudes, behaviours, and ways of relating are shaped by social structures and organizational factors. Finally, we will focus analysis on how the agency of people with dementia is viewed and how bodily expressions of people with dementia along with their socio-cultural contexts are considered in end-of-life decision-making processes.

To analyze documents, we will use critical discourse analysis (CDA) [87, 88]. Similar to Smith’s critical feminist lens [57, 58], CDA is concerned with how discourses are used to exert power and sustain inequities [87]. We will use the principles of CDA [88], viewing discourses as texts that shape how people or events are represented, how social relations are arranged, and how individual and group identities are constructed. We will first examine the sociohistorical context of end-of-life decision-making for LTC residents with dementia, revealing the root of discourses and to whom they are distributed and accessed. Second, we will identify and examine the diversity of the dominant styles and methods of discourses, its meanings to the people involved, and any resistance against controlling attitudes and behaviours [89–91]. After selecting relevant material, we will focus on knowledge, attitudes, ideologies, and norms embedded in dominant (e.g., organizational, governmental, global) discourses and how these exertions of power are related to everyday attitudes and practices. This critical analysis would include examination of the agency of the people and organizations involved during the time period and use of linguistic features (e.g., hyperbole, euphemisms, understatements) [87, 88].

To file and manage data, we will code categories derived from analysis into the computer software program, NVivo. The research team consisting of investigators and graduate research assistants will meet monthly to memo how categories and themes are developed. We will address conflicting interpretations by testing best explanations of participants’ behaviours and decision-making processes with theoretical frameworks, and then, by reaching consensus. As these sensitive qualitative data will be derived from a small urban centre and/or institution, the final dataset (paper and password protected external drive) will be housed at Lakehead University in a locked cabinet accessible only to researchers. Upon reasonable request, we will make available analytical memos to provide evidence of the major findings.

## Discussion

A major strength of this study is the innovative use of theory to guide methods and permit an approach to explore and engage with people living in late stages of dementia in LTC homes about their quality of life. The theory of relational citizenship emphasizes the civic responsibility of institutions and people to provide an optimal environment in which people with dementia and their care partners (of family, LTC home workers) are included in decision-making. This approach provides the basis and means to include the preferences and needs of the person living with advanced dementia. Additionally, a critical feminist lens brings awareness and insight into how gender or other broad structures may lead to stigma and discrimination and shape everyday practices related to end-of-life decision-making for LTC home residents with dementia. Findings from his study have the potential to significantly contribute to future practice and research, as findings may advance theory associated with dementia and end-of-life and shed light on how we can better engage people in later stages of dementia.

This study is the first to explore end-of-life decision-making in LTC homes from the perspective of residents living with dementia. Alongside the underlying theories, use of multiple methods and prolonged engagement will allow a comprehensive and deeper understanding of residents’ perspectives. The interdisciplinary team members of this study have extensive knowledge and experience in engaging with people with dementia and/or at end-of-life and will closely guide and train research assistants in these processes.

Although we have acquired ethics approval from the University, we have experienced challenges from one local LTC home regarding how we acquire consent from residents living with dementia. In keeping with the philosophy of the study to include people living with dementia in decision-making, we offer a layered approach that includes residents with dementia and input from the people involved in their care (staff and family) and that acquires a proxy consent from the substitute family decision-maker as well as assent from the person with dementia. However, one institute requires an objective measure to assess mental capacity of the person with dementia and suggest that researchers ask the person with dementia to describe the study and the benefits and risks of involvement. We believe this request to be unrealistic for people living with advanced dementia in LTC homes. We are attempting to outline protocol for consent that both adheres to the study principles and the institute’s requests.

Overall, knowledge from this study will provide a complex, comprehensive understanding of LTC home end-of-life decision- making practices and thus inform future relational practices and innovative ways of implementing just and inclusive end-of-life decision-making practices for people living with dementia.

### Addendum

A few months into the project, we voluntarily withdrew from the LTC homes when news erupted of a wide-spread pandemic and just prior to the Ontario, Canada ‘lockdown’ policies in which all visitors were restricted from entering LTC homes. Since then, we amended our study protocol and began interviewing family care partners via online Zoom technology asking about end-of-life decisions and experiences pre- and post-COVID-19. We have started to interview paid care partners and wait for safe entry into the LTC homes. Upon returning to LTC homes, we plan to continue interviews (with residents living with dementia and their care partners) and commence ethnographic methods of observation and document review to illuminate dynamic end-of-life decision-making processes.

## Data Availability

The datasets generated and/or analyzed during the current study are not publicly available as they may contain information that could compromise research participant privacy. Data collection for this qualitative study is derived from a small urban city and/or a small institution which may make participants easily identifiable. Additionally, current participant consent forms used for the study do not provide consent for deidentified data to be stored in a public repository. The final dataset will be housed at Lakehead University, Centre for Education and Research on Aging & Health. The dataset (paper files and password protected external drive) will be stored in a locked filing cabinet only accessible to the researchers. Upon reasonable request, the corresponding author will send analytical notes consisting of codes, themes, and quotations that demonstrate the development and reduction of themes.

## Abbreviations

LTC home: long-term care home
ACP: Advance Care Planning
CDA: Critical Discourse Analysis
